# Monthly Summary

**Published:** 1871-07

**Authors:** 


### MONTHLY SUMMARY.

---

*Importance of Right Side of Brain.*—Dr. Brown-Sequard (*London Lancet,*) at the recent meeting of the British Association for the Advancement of Science, reported the result of some interesting experiments on the brains of different animals, tending to show that the right side of the brain was more important for organic life than the left side, and that, although the two sides of the brain were precisely alike when the animals were born, by greater development of the activity of one side it afterwards became quite different from the other. He showed also that epilepsy induced in the guinea-pig could be transmitted to its offspring.—*Med. Times.*

---

*Remedy for Ring-Worm.*—The *L' Union Medicale* contains the following formula for *ring-worm.* ℞—Washed Sulphur, grs. 22: Carb. Potash, grs. 8; Lard ℥ i.—Mix. Continue the application some time after the apparent cure, in order to prevent a return.

*Life-Like in death.*—Rossbach, in a late number of Virchow's *Archiv* (Band 51), has given an interesting account of numerous cases of sudden death on the fields of Beaumont and Sedan, in which the bodies of those killed retained the position and the expression of face present just before death. In one case, a group of six French soldiers were killed by the explosion of a single shell as they were breakfasting in a slight hollow. The shell had struck one of them sitting in the middle full in the back, where it was partly lodged; the fragments had torn away his thighs and buttocks, and killed his comrades. From one of them the skull was carried off, while the face still retained the expression of laughter at the joke of a companion. The next one to him still held delicately raised to his lips, between the forefinger and thumb, a tin cup from which he was about to drink when the explosion had taken off the whole of the upper part of the face and head. The close manner in which they were seated together had prevented the bodies from falling after the lapse of twenty-four hours. In another case a soldier shot through the breast lay half reclining on one side, with the photograph of his wife or lover held up straight before him. Rossbach cannot admit that in such cases the nervous centres must necessarily be injured, nor that death must have been instantaneous. Rigor mortis must have set in between the last moment of life and the first of death.—*Lancet.*

---

*Effects of Alcohol and Tobacco on the Sight.*—On the subject of color-blindness and amblyopia, Dr. Richard H. Derby (*N. Y. Med. Journal*) says: "Almost always both eyes are affected. This form of amblyopia occurs almost solely in men; out of fifty six cases only three were women. It is a disease of adults; its frequency increasing from the twentieth to the fortieth year. In a portion of the cases abuse of alcohol was certainly the cause of the affection, and in others excessive use of tobacco undoubtedly contributed to produce the disease. Forster, in a paper on the injurious action of tobacco on the vision, attaches still greater importance to this agent as a cause of amblyopia, supporting the views of Mackenzie. Sichel, Hutchison, Lureiro, and others. The author cites twenty cases, in which there was a central scotoma, with a horizontal diameter of 18° to 25°, within which large letters could still be recognized. All these patients suffered from some affection of the digestive and nervous system. Loss of appetite, constipation, loss of sleep, were common symptoms. Each one of the twenty patients was a strong smoker, and in eleven of these cases a very marked improvement was observed when the use of tobacco was given up.

---

*The Tolerance of Chloroform.*—Dr. Edward R. Squibb, of Brooklyn, N. Y. (*New York Med. Journal*), remarks that the greatest consumption of chloroform he ever met with was in a patient of Dr. Gustave Morelli, of New York City. This patient was the widow of an Italian physician ; her age 48, and her appearance healthy. She was subject to hereditary migratory gout, the sudden pain of which was so severe that she finally gave up all slower means of temporary alleviation for the prompt action of chloroform. Between the 31st of March and the 16th of December, 1865, a record was kept, and during this time, by Dr. Morelli's direction, she was supplied by Dr. Squibb with fifty-three pounds of purified chloroform. During acute attacks she not unfrequently used two pounds each day.

---

*Nostalgia a Cause of Insanity.*—Dr. Ralph L. Parsons, N. Y. City Lunatic Asylum (*Am. Jour. Insanity*), holds that nostalgia is an important cause of insanity in our foreign population, and in his opinion tends to render the prognosis of those insane from this cause unfavorable. Again, the majority of our Irish patients are of a low order of intelligence, and very many of them have imperfectly developed brains. When such persons become insane, he is inclined to think that the prognosis is peculiarly unfavorable. It is not improbable that an undue proportion of this class of the Irish emigrate to this country.—*Med. Record.*

---

*The Local and Superficial Anæsthetic Effects of the Phenols* — According to Dr. Edward R. Squibb, of Brooklyn, N. Y. (*New York Med. Journal*), the best of these is cresol or cresylic acid, and next phenol or the crystallized carbolic acid. But practically the cheap mixture of the two, called coal-tar creosote or impure carbolic acid, is as good as either. The prompt and complete effect of very diluted aqueous solutions of this creosote upon the pain of burns, erysipelas, &c., led him to infer peculiar anæsthetic properties many years ago, and the numbness or insensibility produced upon the hands by handling it confirmed the idea.

---

*Castor Oil.*—A correspondent suggests that this article of the Pharmacopœia may easily be made palatable by the employment of glycerine as an incipient; in fact the dose will be found as "sweet as honey" and devoid of any unpleasant taste:—

℞. Glycerine (puriss),

Ol. ricini, aa ℥ij.;

Ol cinnamoni, m iv. M.

The ol. cinnam. should be rubbed up with the glycerine, and the ol. ricini then added, and the whole well mixed, by being shaken, when used. In larger doses, lessen the proportion of the glycerine.

---

*Abuse of Alcohol among Women.*—The *Practitioner*, for February, in an article on the use and abuse of alcohol by women, reckons up the amount of spirits habitually consumed by English girls of the middle and upper classes, and finds that in the forms of beer, sherry, champagne, &c., the daily average is from two to three ounces of absolute alcohol. The disastrous consequences of this excess are dwelt upon at length, and the writer concludes by a suggestion that all alcoholic beverages be avoided at evening parties, where, it appears, ladies are at present in the habit of drinking more champagne than the gentlemen.

---

*Fracture of a Rib from Coughing.*—Mr. Miall reports, in the *British Medical Journal*, the case of a woman who, in the seventh month of pregnancy, during a violent fit of coughing, fractured the tenth rib transversely, a little anterior to the tubercle.—*Boston Medical and Surgical Journal.*

---

*Local Application of Carbolic Acid in Diphtheria.*—Dr. C. G. Rothe (*Berliner Klin. Woch.*) applies carbolic acid to the seat of disease by means of a hair-pencil. The patient is to use at the same time, as a gargle, ten or fifteen drops of this agent to a tumbler of water.—*Med. Record.*

*Medical Graduates in the United States.*—The number of medical graduates in the medical colleges of this country for 1871 amounts to about one thousand and fifty, of which New York City gives between three and four hundred.—*Med. Record.*

---

*A Cure for Hemorrhoids.*—Calomel applied dry once or twice a day to tumid and tender hemorrhoids rarely fails to cure them in a few days.—*Boston Medical and Surgical Journal.*—

---

### EDITORIAL DEPARTMENT.

